# Unravelling the connection between COVID-19 and Alzheimer’s disease: a comprehensive review

**DOI:** 10.3389/fnagi.2023.1274452

**Published:** 2024-01-08

**Authors:** Shah Rezlan Shajahan, Suresh Kumar, Muhammad Danial Che Ramli

**Affiliations:** ^1^School of Graduate Studies, Management and Science University, Shah Alam, Selangor, Malaysia; ^2^Faculty of Health and Life Sciences, Management and Science University, Shah Alam, Selangor, Malaysia

**Keywords:** Alzheimer’s disease, dementia, cognition, COVID-19, SARS-CoV-2

## Abstract

Currently, there exists a limited comprehension regarding the correlation between COVID-19 and Alzheimer’s disease (AD). To elucidate the interrelationship and its impact on outcomes, a comprehensive investigation was carried out utilising time-unrestricted searches of reputable databases such as Scopus, PubMed, Web of Science, and Google Scholar. Our objective was to evaluate the impact of various medical conditions on severe COVID-19-related events. We focused on identifying and analysing articles that discussed the clinical characteristics of COVID-19 patients, particularly those pertaining to severe events such as ICU admission, mechanical ventilation, pneumonia, mortality and acute respiratory distress syndrome (ARDS) a serious lung condition that causes low blood oxygen. Through careful data analysis and information gathering, we tried to figure out how likely it was that people with conditions, like AD, would have serious events. Our research investigated potential mechanisms that link AD and COVID-19. The ability of the virus to directly invade the central nervous system and the role of ACE-2 receptors were investigated. Furthermore, the OAS1 gene served as the genetic link between AD and COVID-19. In the context of COVID-19, our findings suggest that individuals with AD may be more susceptible to experiencing severe outcomes. Consequently, it is crucial to provide personalised care and management for this demographic. Further investigation is required to attain a comprehensive comprehension of the intricate correlation between Alzheimer’s disease and COVID-19, as well as its ramifications for patient outcomes.

## 1 Introduction

The World Health Organisation (WHO) recorded more than 6.9 million fatalities as of June 26, 2023, and there were more than 768 million confirmed COVID-19 infections worldwide (Crook et al., 2021). In December 2019, an outbreak of pneumonia with an unknown origin was initially reported in Wuhan, Hubei Province, China (Sharma, 2021). Angiotensin-converting enzyme 2 (ACE-2) receptors are a route by which the virus that causes COVID-19, severe acute respiratory syndrome coronavirus 2 (SARSCoV-2), enters cells (Kumar et al., 2021). SARS-CoV-2 is a positive-strand RNA virus that is exceedingly contagious and calls for exceptional care to stop transmission (Sharma, 2021). Once inside the body, the virus replicates and matures, causing an inflammatory response in some patients, including the activation and infiltration of immune cells by various cytokines (Crook et al., 2021). The ACE-2 receptor is present in many cell types throughout the human body, including the brain, liver, kidneys, spleen, lungs, oral and nasal mucosa, heart, gastrointestinal tract, and arterial and venous endothelial cells (Crook et al., 2021). This indicates how SARS-CoV-2 can harm various organs.

Alzheimer’s disease (AD) is one of the world’s most serious public health challenges, with nearly 10 million new cases diagnosed each year and approximately 50 million people affected worldwide (Esandi et al., 2021). Alzheimer’s disease, in its advanced stages, can also impair a person’s ability to walk and swallow due to widespread brain damage and functional decline. It is also a degenerative condition that decline the patient’s condition on advances stages (Wee and Kumar, n.d.). Alzheimer’s disease is believed to begin at least 20 years before symptoms appear (Alzheimer's Association, 2021). Individuals only notice symptoms like memory loss and language difficulties after years of brain changes (Che Ramli et al., 2022). Symptoms occur because of nerve cell (neurons) damage or destruction in areas of the brain involved in thinking, learning, and memory (cognitive function; Che Ramli et al., 2022). Neurons in other brain areas are also damaged or destroyed as the disease progresses (Alzheimer's Association, 2021). Neurons in areas of the brain that allow a person to walk, and swallow are eventually affected (Alzheimer's Association, 2021).

Multi-system organ failure affecting not just the pulmonary but also the cardiovascular, neurological, and other systems occur in COVID-19 patients (Reiken et al., 2022). According to recent research, COVID-19 may be related to neurodegenerative illnesses (Li et al., 2022). However, it is unclear whether a causal connection exists and how the effect will go (Li et al., 2022). The symptoms of acute cerebrovascular illness were caused by the virus’s direct invasion of the CNS and involuntary SARS-CoV-2 increase in protein and angiotensin-converting enzyme 2 (ACE-2; Ciaccio et al., 2021). These symptoms include confusion, headache, hypogeusia/ageusia, hyposmia/anosmia, dizziness, epilepsy, and acute cerebrovascular illness. During post-mortem examinations, Patients with COVID-19 have SARS-CoV-2 RNA and antigens inside their brain tissue. In COVID-19 pathogenesis, ACE-2 expression is essential. ACE-2 is expressed in neurons, glial cells, endothelial cells, and smooth muscle cells of the arteries in the brain. The temporal lobe and the hippocampus, two cerebral areas involved in the aetiology of Alzheimer’s disease, both express ACE-2 (Ciaccio et al., 2021).

## 2 COVID-19 and Alzheimer’s disease related

Alzheimer’s disease (AD) is acknowledged by the World Health Organisation (WHO) as a global public health concern (Lane et al., 2018). According to Zhang X. X. et al. (2021), AD is the primary cause of dementia and accounts for 50–70% of cases. The virus responsible for the global COVID-19 pandemic is the Severe Acute Respiratory Syndrome Coronavirus 2, commonly referred to as SARS-CoV-2 (Ciaccio et al., 2021). Recent research has demonstrated how COVID-19 affects the CNS and results in neurological problems (Fotuhi et al., 2020). As amyloid beta and neurofibrillary tangles (NFT) are accumulated, the hippocampus, which oversees memory and learning, deteriorates (Rahman et al., 2020). Patients with AD rely on their loved ones and carers to meet their requirements. Managing both Alzheimer’s disease (AD) patients and their caregivers during the COVID-19 pandemic poses significant challenges. The protocols and measures required to manage COVID-19, such as isolation and social distancing, contradict the principles of Alzheimer’s disease management. This incongruence between the two creates complex difficulties for individuals with AD and their caregivers (Rahman et al., 2020).

Additionally, Patients who recovered from severe COVID-19 infection are more likely to acquire stable neuropsychiatric and neurocognitive conditions like depression, obsessive-compulsive disorder, psychosis, Parkinson’s disease, and Alzheimer’s disease. The CNS may be harmed by SARS-CoV-2 directly by the release of neurotoxins or concomitantly through activation of the immune system, which may result in cellular senescence, neurodegeneration, and demyelination (Kumar, 2022). Additionally, research has demonstrated that SARS-CoV-2-infected AD patients had a higher mortality rate. In a study from the Department of Neuroscience at the University of Madrid, 204 participants with Frontotemporal Dementia (FTD) and Alzheimer’s disease (AD) were enrolled. According to the study, 15.2% of these individuals had COVID-19 infection, and sadly, 41.9% of those who had the virus died as a result of their illness (Matias-Guiu et al., 2020).

Based on a recent study, the OAS1 gene is the genetic link between AD and catastrophic COVID-19 results. The study indicates the way oligoadenylate synthetase 1 (OSA1) contributes to a risk factor for AD by enhancing transcriptional networks, which are produced by the microglia (Magusali et al., 2021). By using both animal and human test subjects, they were able to determine that the OSA1 variant, rs1131435, increases the likelihood of acquiring AD (Magusali et al., 2021). Interestingly, this same genetic locus of OAS1 has also been found to have a connection with SARS-CoV-2, the virus causing COVID-19 (Magusali et al., 2021). According to the study, the single nucleotide polymorphism rs1131454(A) and rs4766676(T) are associated with AD, whereas rs10735079(A) and rs6489867(T) are associated with SARS-CoV-2 infections (Magusali et al., 2021). The study shows rs1131454 is inside linkage disequilibrium with newly identified single nucleotide polymorphisms associated with acute COVID-19 infections, suggesting that the spot controls of COVID-19 and AD risk (Magusali et al., 2021). The same study’s functional experiment with human iPSC-derived microglia demonstrated how OSA1 levels control myeloid cells’ pro-inflammatory response to increased interferon levels (Magusali et al., 2021). Therefore, individuals with lowered or impaired levels of OSA1 due to expressive quantitative trait loci (eQTL) variants could demonstrate an inflammatory response to COVID-19 as well as AD-associated pathology, which can trigger a ‘cytokine storm’ and potentially cause cell death and damage to neighbouring cells such as of the alveoli and neurons (Magusali et al., 2021).

In another study, researchers attempt to determine the neurochemical crosstalk between AD and COVID-19. According to studies, during the invasion, SARS-CoV-2 stimulates a neuroinflammatory cascade, astrogliosis, and microglia activation (Rahman et al., 2020). This causes the blood–brain barrier (BBB) to have cooperated due to the inflammation and disrupted homeostasis of the brain (Rahman et al., 2020). Inflammatory mediators are released due to infection of SARS-CoV-2, which is related to a higher BBB permeability and heightened hypoxia (Wu et al., 2020). Acute encephalitis, infectious, toxic encephalopathy, and cerebrovascular attacks (CVAs) occur when the central nervous system (CNS) lacks histocompatibility antigen and predominantly depends on cytotoxic T lymphocytes (Wu et al., 2020). Acute encephalitis symptoms consist of headache and seizures, infectious, toxic encephalopathy symptoms include delirium and coma, as well as a greater risk of CAV caused by a cytokine storm generated by SARS-CoV-2 and coagulation problems (Wu et al., 2020). Additionally, this study examines and demonstrates the relationships between AD and COVID-19 with inflammatory signals such as interleukin 6 (IL-6), interleukin 1 (IL-1), and cytoskeleton-associated protein 4 (CKAP4).

Another link found between AD and COVID-19 is anosmia. The AD symptoms will become more obvious in old age (60 years or more; Mohammad Azizur et al., 2020). However, If AD’s management is managed from an early-stage of Alzheimer’s disease, the progression of these symptoms may be decreased (Mohammad Azizur et al., 2020). Moreover, the “anosmia” novel was an approach to diagnosing AD pathogenesis (Mohammad Azizur et al., 2020). People who carry the e4 allele of apo-lipoprotein E4 (Apo E4) have a higher risk of developing AD and anosmia (Manzo et al., 2021). Anosmia has become one of the most prevalent symptoms of people infected with SARS-CoV-2 (Mohammad Azizur et al., 2020). SARS-CoV-2 uses the ACE2 receptor on the cell’s membrane to enter the cell, and the olfactory tissue has an abundance of ACE2 receptors (Mohammad Azizur et al., 2020). The loss of smell is an early indicator of COVID-19, and for AD, this proves that anosmia is the connection between AD and COVID-19 (Mohammad Azizur et al., 2020).

## 3 Biomarkers of cognition in COVID-19 and Alzheimer’s disease

Restrictions on research due to the recency of COVID-19 have led to the discovery of various cytokines correlating with Alzheimer’s disease. Through genome-wide association studies, it was discovered that ACE2 expression increased in the brain tissue of severely affected Alzheimer’s Disease patients and posed a potential risk for Covid transmission (Ciaccio et al., 2021). This is because increased levels of ACE-2 will constitute an increase the risk of viral entry (Toniolo et al., 2021). Generally, ACE-2 is not just expressed in glial cells, neurons, and arterial and endothelial smooth muscle cells. Still, it is also indicated in the hippocampus and temporal lobe, which are the regions involved in the pathogenesis of AD (Ciaccio et al., 2021). Other than this biomarker, the apolipoprotein E ε4 genotype is also another important association between AD and Covid-19, as it is both a biomarker for Covid-19 increased severity and a genetic risk factor for late-onset AD (Frontera et al., 2022). Polymorphism APOE ε4 will also increase the risk of AD when the homozygous genotype of ε4 is associated with a 14-fold (Ciaccio et al., 2021). Those who are homozygous for APOE 4 have also demonstrated a greater prevalence of SARS-CoV-2 infection. According to Kuo (2020), APOE ε4 can increase the vulnerability to neurodegeneration and viral infection in our body. So, this will postulate SARS-CoV-2 infection in individuals with susceptible genetic variants (Ciaccio et al., 2021). The recent study done by UK-Biobank which has shown that APOE ε4 homozygotes have a 2.2-fold increased risk for the infection of Covid-19, and if it is a 4.3-fold case, it could turn into a fatal case (Toniolo et al., 2021).

Furthermore, systemic inflammation will activate astrocytes and microglia, which will then help to secrete pro-inflammatory cytokines, including IL-6, IL-1β, IL-12, and TNF-α. These biomarkers can cause synaptic dysfunction, potentially leading to AD, and inflammatory biomarkers like IL-6, galectin-3 (Gal-3), and IL-1 have been showing a linkage between AD and Covid-19 (Ciaccio et al., 2021). IL-6 represents not just a prognostic biomarker that is reliable in SARS-CoV-2 infection but also in AD. When the levels of IL-6 are increased, it will progress to AD and lead to worse cognitive performance, and this will cause a higher risk of developing severe Covid-19 and mortality (Ciaccio et al., 2021). When IL-6 interacts with IL-6R to exert biological effects, it could be soluble or expressed in the epithelial cells, immune membrane, and liver cells (Ciaccio et al., 2021). In the same way, glial cells and neurons produce the pro-inflammatory cytokine IL-1 (Ciaccio et al., 2021). In the brains of individuals affected by both Alzheimer’s disease (AD) and COVID-19, there is an observed increase in the levels of IL-1 (Interleukin-1; Ciaccio et al., 2021). Since it is involved in the regulation of memory processes physiologically and physiologically regulation of hippocampal plasticity, Covid-19 patients may enhance cognitive decline, and this will lead them to develop AD in the future (Ciaccio et al., 2021).

In addition to these biomarkers, transforming the growth factor of beta 1 (TGFB1), a vascular cell adhesion protein 1 (VCAM1), and ras-related protein Rab-7a (RAB7A) are the additional biomarkers that have demonstrated a connection between AD and Covid-19 (Zhou et al., 2021). The blood expression level of an individual is favourably correlated with their performance on the high memory test, making RAB7A a potential biomarker for AD (Zhou et al., 2021). It is also a top host factor in SARS-CoV-2 datasets based on the CRISPR-Cas9 and a direct target of SARS-CoV-2’s non-structural protein 7 (nsp7), which will aid in lowering SARS-CoV-2 entry into cells. VCAM1 is a target for treating age-related neurodegeneration since it is connected to changes in the white matter’s structure and the severity of dementia (Zhou et al., 2021). In contrast to moderate individuals, significantly increased serum levels of VCAM were found in patients with severe COVID-19. Then there is TGFB1, a cytokine that regulates cell differentiation and growth. Through the analysis of a substantial dataset of RNA sequencing data obtained from peripheral blood mononuclear cells of individuals diagnosed with COVID-19, researchers discovered a noteworthy observation. The expression of TGFB1 (Transforming Growth Factor Beta 1) was found to be significantly reduced in patients with mild COVID-19 symptoms as well as those who required intensive care unit (ICU) level care, in comparison to individuals who were not affected by COVID-19 (Zhou et al., 2021). The infection of SARS-CoV-2 led to alterations in various Alzheimer’s disease (AD) markers within peripheral blood mononuclear cells (PBMCs). These changes affected several proteins, including SERTA domain-containing protein 3 (SERTAD3), TGFB1, kinase D-interacting substrate of 220 kDa (KIDINS220), glutathione S-transferase M3 (GSTM3), arylsulfatase B (ARSB), insulin-like growth factor 1 (IGF1), and natural killer tumour recognition (Zhou et al., 2021). Among both Alzheimer’s disease (AD) and COVID-19 patients, certain biomarkers exhibited changes in a similar direction, while others displayed alterations in a different direction. Notably, the expression of TNF receptor superfamily member 1B (TNFRSF1B) consistently showed changes in cerebrospinal fluid (CSF) samples obtained from patients affected by both COVID-19 and AD (Zhou et al., 2021). In a recent study, researchers have identified and chosen three cerebrospinal fluid (CSF) markers, TNFRSF1B, CXCL10, and SPP1, along with three blood markers associated with Alzheimer’s disease (AD), which are GSTM3, TGFB1, and NKTR. These markers have been specifically selected for their relevance and potential implications in understanding AD (Zhou et al., 2021). The findings of the recent study reveal that NKTR exhibits interactions with several host factors involved in SARS-CoV-1 and SARS-CoV-2, such as zinc finger CCCH-type containing 18 (ZC3H18), MERS-CoV, as well as casein kinase II subunit alpha (CSNK2A2). This highlights the potential significance of NKTR in the context of these viral infections (Zhou et al., 2021). These have demonstrated that SARS-CoV-2 infection would change some of the expression for the chosen AD markers, impacting various immune-related genes and perhaps causing the patients to experience neurologic impairment resembling AD (Zhou et al., 2021).

**Figure 1:**
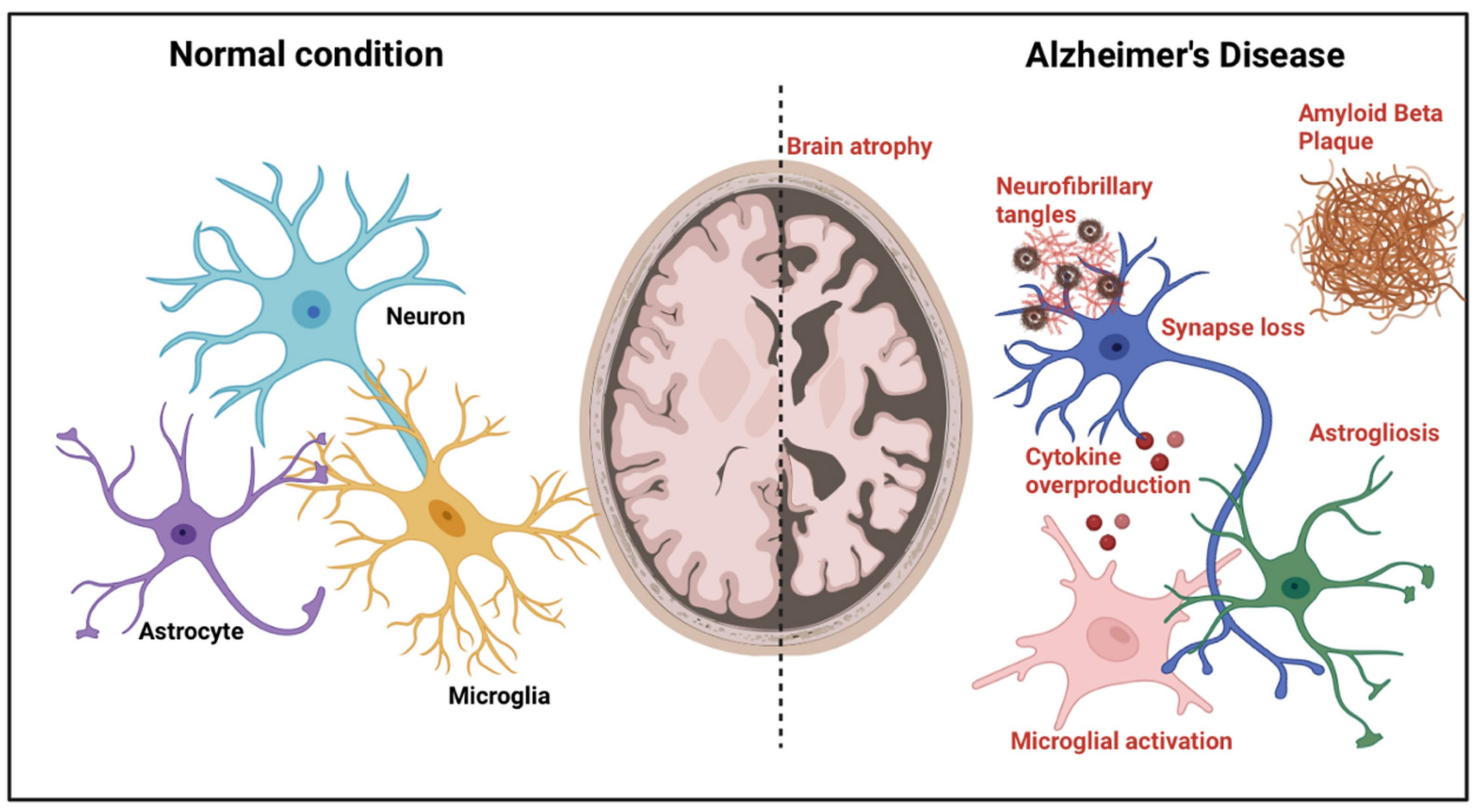
Alzheimer’s disease pathological features. A diagram depicting the pathogenic alterations in AD brains compared to normal brains. Brain atrophy caused by neuronal loss is visible at the gross anatomical level. At the microscopic level, toxic amyloid-β, intraneuronal neurofibrillary tangle development, synaptic loss, activation of microglia, astrogliosis, cytokine overproduction, and neurite dystrophy are detected.

Besides, evidence suggests that Aβ peptides are one of the factors that increase the risks in Covid-19 patients to get diagnosed with AD, which these peptides act as antimicrobial peptides (Ciaccio et al., 2021). Loss of pericytes and endothelial dysfunction will reduce the cerebral metabolites’ clearance, including Aβ peptides (Ciaccio et al., 2021). As a result, there will be an accumulation and excess of Aβ protein in the senile plaques, especially in the hippocampus, representing the main pathophysiological mechanism underlying AD (Ciaccio et al., 2021). To diagnose of AD depends on the detection that we get from the CSF biomarker profile, which are the ratio Aβ 1–42/1–40, decrease in amyloid beta 1–42 (Aβ 1–42), and the increase of p-Tau and t-Tau levels (Ciaccio et al., 2021). T-tau protein is a biomarker of neuronal damage or death, and according to Ciaccio et al., some of the authors have found that there was an increase of CSF t-Tau levels in COVID-19 patients, in which the increased levels of this biomarker will cause several neurodegenerative diseases, including AD (See Figure 1; Ciaccio et al., 2021). Furthermore, in severe COVID-19 patients, it is found to have increased levels of Gal-3, and this will progress COVID-19 due to lung fibrosis and hyper-inflammation reaction (Ciaccio et al., 2021). In AD patients’ serum, it has also been found to have increased levels of Gal-3 (Ciaccio et al., 2021). Gal-3, a lectin-family member that binds carbohydrates, is crucial for pathological and physiological processes like fibrosis and inflammation (Ciaccio et al., 2021). Gal-3 is thought to be involved in aggregation and the production of amyloid plaques. Therefore, the elevated levels in Covid-19 patients may harm them and eventually result in the development of AD (Ciaccio et al., 2021).

## 4 Prevention and treatment

According to the World Health Organisation (WHO), the second-highest neurological disorder that affects people worldwide happens to be Alzheimer’s disease (AD). Due to this, many of these Alzheimer’s patients may face various conditions or complications if they get affected by the COVID-19 coronavirus. In terms of prevention and treatment, no outright cure is known for AD. Still, there are several ways to slow down the progression of Alzheimer’s in patients affected by COVID-19 since it is known to speed up AD progression. There are also ways to reduce the risk of an elderly patient with COVID-19 from developing Alzheimer’s. Firstly, anti-inflammatory therapy can be carried out. According to Wang et al. (2021), Systemic inflammation from COVID-19 and AD is brought on by a rise in TNF- and other pro-inflammatory chemicals. Studies on anti-inflammatory treatments conducted in mice, rats, and monkeys demonstrate favourable outcomes (Wang et al., 2021). It has been demonstrated that using TNF inhibitors as a treatment reduces neurofibrillary tangles, amyloid precursor protein, and amyloid beta (A) plaques and functions as an important immune modulator that aids in the prevention of AD (Wang et al., 2021). In addition to TNF inhibitors, a different study discovered that long-term usage of non-steroidal anti-inflammatory medicines could suppress the microglial activation brought on by A oligomers that cause neuronal ectopic cell cycle events, which will aid in preventing the onset of AD (Wang et al., 2021).

Next, another method that can be used is antioxidant therapy. Antioxidant therapies can help Alzheimer’s patients with COVID-19 as both these diseases are known to increase oxidative stress on the human body (Wang et al., 2021). Certain antioxidants, such as vitamin E and its derivatives, were shown to mitigate oxidative stress and mitochondrial dysfunction in brain cells (Wang et al., 2021).

Anticholinesterase Inhibitors and Memantine are frequently used in COVID-19 Infected Alzheimer’s Patients (Brown et al., 2020). Anticholinesterase inhibitors, which are also known as cholinesterase inhibitors, are a type of drug that will reduce the breakdown of acetylcholine, which is a neurotransmitter that is found in the brain and the nervous system. As for memantine, it is a medication that helps to slow down the progression of AD. For dementia (BPSD), behavioural and psychological symptoms that could compromise isolation efforts, such as motor agitation, intrusiveness, or wandering, medications such as antidepressants, antiepileptics, and other psychotropic medications are commonly used as well (Brown et al., 2020). For COVID-19, some potential drugs such as hydroxychloroquine (HCQ) and chloroquine (CQ) could interact with the cholinesterase inhibitors, which could lead to the alteration of the pharmacological function of the cholinesterase functions (Xia et al., 2021). It can increase the chance of toxic side effects and adverse events such as bradycardia, gastrointestinal issues, falls, fractures, heart attacks, or strokes (Balli et al., 2020).

Nutritional intervention is also another method that was also recently found to be useful in slowing down AD progression and prevention as well. Nutritional interventions were shown to reduce the rate of occurrence and severity of AD through modulation of the flora of the gut and cerebral Aβ production (Wang et al., 2021). Prebiotics, like wheat bran, has been shown to reduce neuroinflammation, encourage the growth of commensal bacteria, positively influence the gut-brain axis, and delay the onset and progression of AD (Wang et al., 2021). Other than nutritional interventions, physical exercise is also a viable option as it is shown to prevent several AD risk factors. Physical exercise’s main benefit will be the anti-inflammatory and antioxidative effects and greater cerebral blood flow (Wang et al., 2021).

Another method that may be effective may be controlling blood glucose levels. Both AD and diabetes mellitus have the same pathophysiological factors, such as severe inflammation, oxidative stress, and dysfunction of the mitochondria (Wang et al., 2021). COVID-19 can cause the development of dysglycemia and possibly type 1 Diabetes Mellitus (Wang et al., 2021). As a result, AD may subsequently develop because of this. Fiore et al. (2019) demonstrate how hyperglycemia and hypoglycemia can result in cognitive impairment and AD. Hence, methods for controlling blood glucose levels may positively benefit Alzheimer’s patients with COVID-19 by minimising the risk of AD or slowing down its progression.

## 5 COVID-19 long-term effects on Alzheimer’s disease patient

Elderly people with Alzheimer’s disease and other dementias are more vulnerable to the COVID-19 pandemic (Palmieri et al., 2020). Research by Luigi et al. has carried out a study to examine symptoms and pre-existing comorbidities by analysing clinical reports of COVID-19 deaths. The results suggested comorbidities are related to a higher mortality risk and negative consequences in COVID-19 patients (Palmieri et al., 2020). Moreover, it is shown that SARS-CoV-2 can damage the peripheral and the central nervous system through both direct and indirect pathways, potentially leaving COVID-19 patients at higher risks for neurological difficulties, including depression, Parkinson’s disease, AD, etc., after recovering from severe symptoms (Palmieri et al., 2020). Severe symptoms of COVID-19 in patients with pre-existing dementia can be explained through several reasons (Mok et al., 2020). Firstly, demented patients are prone to have a high viral load as it is comparatively more difficult for them to understand the importance of standard operating procedures (SOPs) and to comply with public health measures (Mok et al., 2020). Second, the important population of AD patients takes residence in care homes where the infection is likely to spread (Mok et al., 2020). Lastly, COVID-19 causes a secondary effect on underlying brain pathologies as SARS-CoV-2 has been shown to trigger or accelerate neurodegeneration processes that possibly explain long-term neurodegenerative effects in the elderly population (Vincent).

### 5.1 Non-neurological effects of COVID-19 on AD patients

In response to the impact of COVID-19 in 2020, governments worldwide acted promptly by implementing various public health measures. These measures included the enforcement of movement restriction orders, mandatory wearing of face masks in public settings, and the promotion of social distancing to mitigate the spread of the virus (Vincent, 2020). During this period, people with cognitive impairments such as dementia or AD may experience greater stress and anxiety due to sudden changes in the environment and people’s behaviour (Vincent, 2020). Movement controls during quarantine cause AD patients to be restricted to confined spaces which can further lead to depression or apathy (Palmieri et al., 2020). The pandemic and quarantine also cause AD patients to be isolated in hospital environments or care homes, away from their family and friends, increasing the risk for further dementia-related decline (Palmieri et al., 2020). It is also significantly harder for AD patients to comprehend and execute defensive measures such as wearing face masks and sanitising frequently (Mok et al., 2020). Patients with agitation and wandering conditions are exposed to higher risks of infection (Mok et al., 2020).

Furthermore, physical distancing does not apply to AD patients as they depend on caregivers to carry out daily tasks such as bathing. In contrast, some patients reside in care houses where patients live close to other people (Mok et al., 2020). This makes them more vulnerable to infection than non-demented people (Palmieri et al., 2020). AD patients are usually diagnosed with age-related sensory deficits and perceptual troubles (Gil and Arroyo-Anlló, 2021). This leads to prosopagnosia, commonly known as face blindness or the inability to recognise other people through their morphological structures of faces (Gil and Arroyo-Anlló, 2021). Wearing face masks during the pandemic has become a common practice. Still, it increases the difficulty for AD patients to recognise their family members and friends as the face is partially concealed (Gil and Arroyo-Anlló, 2021). Fragmented perception of the face also reduces the ability of AD patients to discriminate facial emotions (Gil and Arroyo-Anlló, 2021).

### 5.2 Neurological effects of COVID-19 on AD patients

Studies have shown that COVID-19 will likely leave long-term neurological complications in patients who survive and recover from the infection (Chen et al., 2022). Meanwhile, neuroinflammation is suggested to be the underlying cause of neurological complications and the important bridge between COVID-19 and AD (Finelli, 2021; Chen et al., 2022). There are a few potential mechanisms that increase the risk of developing long-term neurological consequences, leading to the acceleration of pre-existing AD progression or initiating AD development in COVID-19 patients (Iodice et al., 2021). These potential mechanisms include Renin-angiotensin system (RAS) hyperactivation, systemic inflammation, and damage to the CNS by direct viral infection (Heneka et al., 2020).

COVID-19 neurological effects research has highlighted a need for a better knowledge of how the virus may damage brain health and may contribute to the advancement of Alzheimer’s disease. Table 1 shows recent clinical investigations reveal that people with Alzheimer’s disease may be more likely to have serious problems if they contract COVID-19.

**Table 1:** Clinical findings between alzheimer’s disease and COVID-19.

Type of COVID-19 sample	Clinical findings	References
A study conducted among the UK Biobank community.	-Delirium being a leading symptom -Mortality rate was higher among patients	Rudnicka-Drożak et al. (2023)
COVID-19 patients with ARDS and neurological manifestations admitted to an ICU.	-Prevalence of cognitive impairment: 100%	Chaumont et al. (2020)
Patients admitted to an ICU with ARDS due to COVID-19.	-Prevalence of dysexecutive syndrome at discharge: 36%	Helms et al. (2020)
COVID-19 hospitalised patients admitted to a neurology unit or with neurological symptoms.	-Prevalence of short-term memory loss: 24%	Pinna et al. (2020)
CoroNerve Platform COVID-19 hospitalised patients with neurological manifestations.	-Prevalence of neurocognitive disorder: 4.8%	Varatharaj et al. (2020)

ARDS, Acute respiratory distress syndrome; ICU, Intensive care unit; UK, United Kingdom.

#### 5.2.1 Renin-angiotensin system hyperactivation

The renin-angiotensin system (RAS) remains a hormone system that regulates blood pressure, electrolyte balance, and vascular resistance (Wang et al., 2021). The RAS contained in the pathogenicity of COVID-19 is angiotensin-converting enzyme 2 (ACE2), which acts as a critical point for SARS-CoV-2 and belongs to part of this system (Sarzani et al). Within the RAS, ACE2 regulates blood pressure by converting angiotensin 2 (Ang-II) into angiotensin (Ang), thus inhibiting the RAS pathway (Wang et al., 2021). During an infection, ACE2 binds to the protein of spike from SARS-CoV-2, causing a reduction in enzymatic function (Wang et al., 2021). As a result, Ang-II levels are increased, disturbing the signalling pathway in RAS and promoting brain degeneration (Wang et al., 2021). Moreover, the binding of the SARS-CoV-2 and ACE2 triggers the formation of a cytokine storm, characterised by increasing stages of IL-1, IL-6, and TNF (El-Arif et al., 2021). Another way through which SARS-CoV-2 can cause RAS hyperactivity in the brain through stimulating the production of neurotoxins and proinflammatory factors (Wang et al., 2021). RAS hyperactivation caused by elevated Ang-II leads to microglial inflammatory response and oxidative stress, favouring AD development (Wang et al., 2021). Ang-II activates NLRP3 inflammasome, which, as mentioned, is a key mediator in AD (Miners et al., 2020). Meanwhile, RAS hyperactivation also impairs Aβ clearance (Miners et al., 2020). These factors altogether contribute to AD development (Miners et al., 2020). However, ACE2 expression and the consequence on AD pathology remains controversial and requires support from further studies (Chen et al., 2022).

#### 5.2.2 Systemic inflammation

Severe systemic inflammation caused by SARS-CoV-2 is predicted to have long-term negative consequences like cognitive impairment (Finelli, 2021). SARS-CoV-2 infection causes immune system dysfunction, which can lead to suppression of neurogenesis, synaptic damage, and neuronal death, all of which are associated with the aetiology of Alzheimer’s disease (Chen et al., 2022).

##### 5.2.2.1 Cytokine storm

Cytokines are messenger molecules immune cells produce (Rahman et al., 2020). It alters the function of proteins and changes the gene expression of receptor molecules (Ragab et al., 2020). Proinflammatory cytokines endorse inflammation, while anti-inflammatory cytokines decrease inflammation (Ragab et al., 2020). Further, cytokines are categorised based on their functions into 4 major groups, which are Interleukins (IL), Tumour Necrosis Factor (TNF), and Interferons and Colony Stimulating factors (CSF; Ragab et al., 2020).

During an infection, the invasion of SARS-CoV-2 stimulates humoral and cell-mediated immunity to battle infection (Hu et al., 2021). However, a deadly and uncontrolled inflammatory response known as the ‘cytokine storm’ can lead to the fast secretion of pro-inflammatory cytokines, creating an inequity between pro-inflammatory cytokines and anti-inflammatory cytokines (Ragab et al., 2020). The cytokine storm increases vascular permeability and abnormal blood coagulation. It explains the multi-organ damage found in COVID-19 patients (Wang et al., 2020), as the effects of SARS-CoV-2 in CNS also explained as the cytokines can attack brain regions, resulting in harm to healthy neurons (Hardan et al., 2021). Moreover, Proinflammatory cytokines like interleukin (IL)-1β, IL-17, IL-6, also tumour necrosis factor-α (TNF-α) that are involved in AD development are significantly elevated in cerebrospinal fluid of COVID-19 patients (Finelli, 2021; Wang et al., 2021). High levels of IL-1β decrease hippocampal neurogenesis and increase apoptosis (Boldrini et al., 2021) IL-17 targets neutrophils, promoting inflammation and brain tissue damage (Wang et al., 2021). TNF-α links peripheral and central inflammation and modulates neuropathological mechanisms in AD. In contrast, higher stages in COVID-19 individuals are associated with a higher percentage of cognitive impairment months after infection (Finelli, 2021). Elevated levels of IL-6 are related to hippocampus shrinkage and decreased human cognitive performance, one of the early symptoms of AD (Mohammad Azizur et al., 2020). Acknowledge a nucleic acid, which contains amyloid fibrils, Type I interferons (IFN) mediate inflammation, ultimately leading to synaptic loss, an effect that is strongly related to cognitive decline (Hardan et al., 2021). Through these mechanisms, proinflammatory cytokines play a significant part in AD progression (Wang et al., 2021).

##### 5.2.2.2 NLRP3

Inflammasomes are multiprotein complexes that build or activate cytokines during an inflammatory response. An inflammasome called NLRP3 (NOD-, LRR- and pyrin domain-containing protein 3) controls inflammatory signalling and the release of proinflammatory cytokines IL-1 and IL-18 (Wang et al., 2021). Initiating caspase-1 self-cleavage to create active caspase-1 when NLRP3 is activated is crucial for the maturation of IL-1 and IL-18 (Wang et al., 2021). Inflammasome activation may occasionally result in cell death (Mark et al., 2021). Although inflammasomes are beneficial immune responses to infections, at the same time, they might also cause collateral damage to recipient cells due to hyperinflammatory responses and are also known to be the key mediator of AD development. (Ding et al., 2018 Mark et al., 2021) In a COVID-19 patient, overstimulation in the NLRP3 inflammasome pathway leads to a systemic inflammatory response during infection (Wang et al., 2021). Consequently, the impairment of useful immune functions in the brain is caused by the NLRP3 inflammasome-driven inflammation and aberrant accumulations of peptides linked to neurodegeneration, such as fibrillar amyloid (Finelli, 2021). A work by Ising et al. (2019) showing that NLRP3 inflammasome produces hyperphosphorylation and aggregation and is crucial for the emergence and advancement of beta-amyloid pathology in mice supports this. Additionally, it has been demonstrated that NLRP3 inflammasome activation encourages tau pathology, which speeds up the development of AD (Chen et al., 2022).

Through both direct and indirect processes, including ARDS, hypercapnia, and ORF3a protein-mediated activation, SARS-CoV-2 has been exposed to raise NLRP3 levels (Ren et al., 2020). In the study by Ren et al. (2020), NLRP3 inflammasome was discovered for the first time in similarly stimulated epithelial cells infected with SARS-CoV-2. Furthermore, the SARS-CoV genome encodes three ion channel proteins, namely ORF3a, ORF8a, and E (Ren et al., 2020). It is noteworthy that ORF3a plays a role in virus-host interactions. The secretion of the proinflammatory cytokine IL-1 and the activation of the NLRP3 inflammasome by the SARS-CoV-2 ORF3a protein leads to the demise of respiratory tract epithelial cells (Xu et al., 2022). This conclusion is further supported by the observation that lung alveolar epithelial cells obtained from autopsy samples of COVID-19 patients who passed away exhibit significant activity of the NLRP3 inflammasomes (Toldo et al., 2021).

Acute respiratory distress syndrome is a common complication in severe COVID-19 patients, and it is caused by dysregulated hyperinflammation caused by SARS-CoV-2 (Wang et al., 2021). Invasion of the virus stimulates innate immune response and activates NLRP3 inflammasome (Wang et al., 2021). The NLRP3 inflammasome then functions to mediate lung inflammation in SARS-CoV-2 infection. As stated, SARS-CoV-2 targets ACE2 receptors found on type II alveolar epithelial cells, causing them to undergo cell apoptosis (Mark et al., 2021). Signals of cellular damage or stress released can lead to NLRP3 inflammasome activation (Mark et al., 2021). Activation stimuli of NLRP3 inflammasome include foreign matter, extracellular adenosine triphosphate (ATP), toxins, and mitochondrial processes (Mark et al., 2021). During an infection, alveolar macrophages initiate a proinflammatory response which triggers the secretion of cytokines like TNF-α and IL-1β (Freeman and Swartz, 2020). Secretion of these cytokines induces a widespread NLRP3 activation to form a proinflammatory positive feedback cascade (Freeman and Swartz, 2020). Hypercapnia, defined by high levels of arterial carbon dioxide, is a side effect caused by protective lung ventilatory strategies used to treat severe ARDS patients (Freeman and Swartz, 2020). However, it is shown that hypercapnia can also activate NLRP3 inflammasomes to induce IL-1β overproduction, which is linked to neuroinflammation, increased neuronal cell death, and contributes to the pathogenesis of cognitive impairments (Freeman and Swartz, 2020).

##### 5.2.2.3 Oxidative stress

Reactive oxygen species (ROS) encompass a group of chemically reactive oxygen-containing molecules, including peroxides, superoxide, hydroxyl radicals, and more, that are produced during instances of inflammation (Ionescu-Tucker and Cotman, 2021). The existence of an unpaired valence electron makes ROS free radicals attack the different cells and damage DNA, proteins, and lipids (Ionescu-Tucker and Cotman, 2021). In contrast, antioxidants are chemicals that lessen the effect of free radicals (Wang et al., 2021). An imbalance in the amount of ROS and antioxidants within the human body causes oxidative stress, which plays a part in both the pathogenesis of SARS-CoV-2 infection and AD (Wang et al., 2021). This suggests that SARS-CoV-2 might contribute to AD through an oxidative stress mechanism (Wang et al., 2021).

During SARS-CoV-2 infection, angiotensin-converting enzyme 2 (ACE2) binds its viral protein, increasing Ang-II (Kumar et al., 2022). As Ang II acts as an oxidative stress enhancer, the increased presence of Ang-II promotes the presence of ROS and creates oxidative stress in the body (Meinhardt et al., 2021). In comparison, decreased ACE2 is also related to the production of ROS in CNS (Meinhardt et al., 2021). ROS are harmful as they can cause lipid peroxidation and mitochondrial dysfunction (Meinhardt et al., 2021). To gain stability, ROS can donate an electron to a nearby lipid molecule from the phospholipid bilayer (Butterfield, 2020). This causes the lipid molecules to become reactive and initiate a chain reaction that results in cell membrane damage and lysis (Butterfield, 2020). In addition, oxidative damage is a major character in the brain of AD patients. At the same time, studies suggest that lipid peroxidation is the first type of oxidative damage associated with amyloid β (Aβ). This amino acid is critical to the pathophysiology of AD (Butterfield, 2020).

In the mitochondria, superoxide radicals are created by-products of the electron transport chain (Ionescu-Tucker and Cotman, 2021) naturally. Excessive mitochondrial ROS can cause damage to the electron transport chain, subsequent in increased generation of superoxide radicals in a positive feedback cycle (Ionescu-Tucker and Cotman, 2021). Frequent damage to the mitochondria eventually results in degradation (Ionescu-Tucker and Cotman, 2021). Significantly, dysfunctional mitochondria are one of the first markers and a vital cause of Alzheimer’s disease (Ionescu-Tucker and Cotman, 2021). Loss of mitochondrial function interferes with the expression and processing of amyloid precursor protein (APP) and facilitates the formation of beta-amyloid plaques (Ionescu-Tucker and Cotman, 2021). This relationship further demonstrates the possible role of the virus in the expansion of AD and other associated neurodegenerative diseases (Ionescu-Tucker and Cotman, 2021).

#### 5.2.3 Damage to the CNS by a direct viral infection

According to studies, SARS-CoV-2 can penetrate the CNS and cause neuroinflammation and neuronal damage, which aids in the emergence of neurodegenerative disorders like Alzheimer’s disease (Wang et al., 2020). The blood–brain barrier (BBB), olfactory nerve channels, and trans-synaptic routes are possible routes by which SARS-CoV-2 could enter the brain (Zhang Q. et al., 2021).

**Figure 2:**
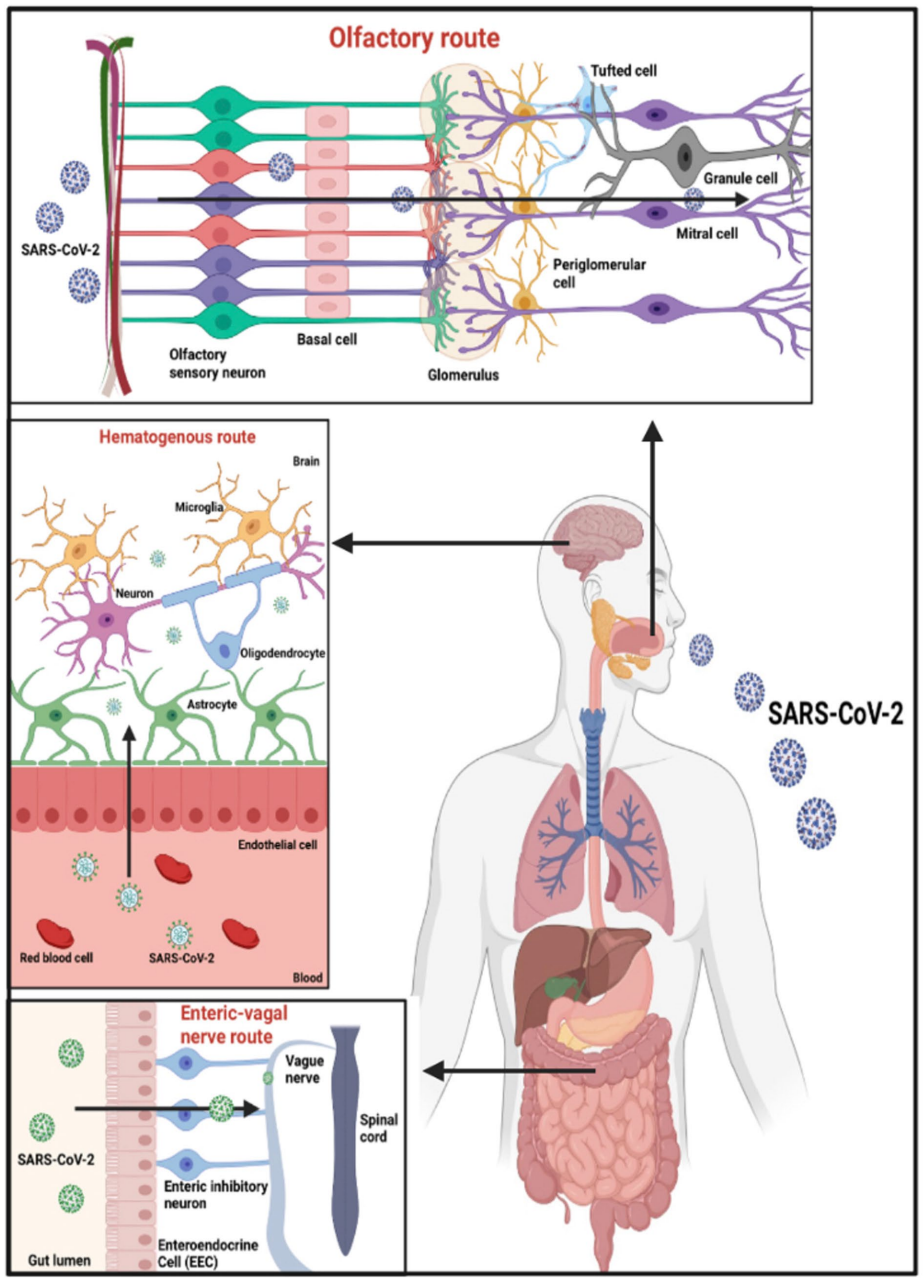
Potential entry points for SARS-CoV-2 entering the CNS. Based on the tissue/cell expression patterns of the viral binding receptor ACE2 and cell entry-associated proteases TMPRSS2 on the olfactory epithelium, myelin-forming cells, enteric neurons, and vascular endothelium, it is possible that SARS-CoV-2 enters the human blood–brain barrier through the neural route of myeline sheaths of olfactory, enteric, and vagal nerves, as well as the hematogen.

A complex system called the Blood–Brain Barrier (BBB) encircles most of the brain’s blood vessels (kadry et al). By serving as a barrier between the bloodstream and the brain’s extracellular space, it regulates the flow of substances between them (Zhang Q. et al., 2021). The BBB protects most blood vessels in the brain, except for blood vessels in circumventricular organs (CVO), as their function requires access to the bloodstream (Zhang Q. et al., 2021). Recent research has shown that SARS-CoV-2 can infect ACE2 receptors on the vascular endothelium of the BBB, allowing blood material to reach the brain (Zhang Q. et al., 2021). By causing instability or by way of monocytes, the activation of inflammatory cytokines might raise the BBB’s permeability and allow the entry of cytokines and SARS-CoV-2 into the brain parenchyma (Zhang Q. et al., 2021) as shown in Figure 2.

Reactive astrogliosis, microglial activation, and a neuroinflammatory cascade are brought on by SARS-CoV-2 upon invasion (Iodice et al., 2021). Disruption of brain homeostasis and neuronal death damages the function of BBB, leading to long-term neuropsychiatric consequences (Iodice et al., 2021). Furthermore, the virus can reach brain tissue via CVOs and has been found in brain vascular endothelium (Boldrini et al., 2021). CVO and brain stem viral infection explains COVID symptoms such as ageusia, nausea, and vomiting, as also autonomic abnormalities, and anxiety (Boldrini et al., 2021). Due to the penetration of blood content, viral particles can enter and damage neurons directly as ACE2 receptors are present (Majid et al., 2021). In addition, recent studies have shown that immune cells that express ACE2 receptors, such as lymphocytes, granulocytes, monocytes, and monocyte derivatives, provide another pathway for entering through the BBB through a trojan horse mechanism (Wang et al., 2020). The SARS-CoV-2 will enter the cytoplasm of these immune cells and cross the BBB to access the CNS for replication (Pezzini and Padovani, 2020).

Several types of research have shown trans-synaptic transfer through peripheral nerve terminals (Pezzini and Padovani, 2020). By binding to ACE2 receptors on peripheral neurons, it can spread through the axonal endoplasmic reticulum over large distances (Fenrich et al., 2020). Additionally, SARS-CoV-2 can penetrate the CNS via the olfactory route, which explains the loss of smell in COVID-19 patients (Meinhardt et al., 2021; Chen et al., 2022). As a part of the respiratory system, the olfactory mucosa is in the upper region of the nasal cavity (Wang et al., 2020). The olfactory epithelium consists of 10–20 million olfactory receptor neurons that detect the sense of smell (Wang et al., 2020). Unsurprisingly, the entry proteins ACE2 and TMPRSS2 involved in the binding of SARS-CoV-2 are found in abundance in these olfactory receptor neurons (Wang et al., 2020). Following infection, the virus binds to ACE2 receptors on olfactory receptor neurons, spreads through the olfactory bulb, and eventually reaches the hippocampus and other brain structures (Pezzini and Padovani, 2020). Notably, the hippocampus is one of the brain areas affected during the early stages of AD progression (Iodice et al., 2021).

Regardless of the pathway used by SARS-CoV-2, it enters the CNS and initiates viral replication, resulting in neuronal cell death and immune system activation in the brain, which may explain the acute symptoms of COVID-19 and long-term complications of SARS-CoV-2 infection in Alzheimer’s disease patients (Wang et al., 2020).

### 5.3 The comparison of AD patients with COVID-19 and AD patients without COVID-19

Based on the selected reviews, we have compiled a table (Table 2) that compares AD patients with COVID-19 to AD patients without COVID-19. According to a study by Frontera et al. (2022), hospitalised COVID-19 patients exhibited greater levels of neurodegenerative biomarkers than non-COVID subjects with normal cognition, mild cognitive impairment, or Alzheimer’s dementia. The results showed that various neurodegenerative biomarkers, including t-tau, p-tau181, UCHL1, GFAP, and NfL, were raised in hospitalised COVID-19 patients. The Table 2 provides an overview of various biomarkers and pathological features in Alzheimer’s disease (AD) patients with and without COVID-19 infection. The findings suggest that COVID-19 infection has a significant impact on the pathogenesis of AD, leading to alterations in inflammatory responses, oxidative stress, ACE2 functions, and neurodegenerative biomarkers. Notably, the presence of COVID-19 in AD patients appears to exacerbate some of the AD-related pathological changes, such as increased proinflammatory cytokines, NLRP3 activation, and oxidative stress. Additionally, direct viral infection in AD patients with COVID-19 leads to damage to the central nervous system, which can accelerate AD progression. These observations highlight the importance of monitoring AD patients who contract COVID-19 and considering potential implications for disease management and intervention strategies. Further research is needed to elucidate the underlying mechanisms and long-term consequences of COVID-19 on AD pathology.

**Table 2:** Comparison of AD patients with and without COVID-19 based on relevant biomarkers and pathological features.

	AD patients with COVID-19	AD patients without COVID-19	
Proinflammatory cytokines	Cytokine storm greatly increases number of proinflammatory cytokines such as IL-6 and TNF-alpha.	no significant difference in cytokine levels	Frontera et al. (2022)
NLRP3	SARS-CoV-2 induces activation of NLRP3, increasing NLRP3 levels.	no significant difference in NLRP3 levels	Ding et al. (2018)
ACE2	ACE2 enzymatic functions is reduced.	Functions and number of ACE2 unaffected	Ding et al. (2018)
Oxidative stress	Higher	Lower	Suhail et al. (2020)
Damage to the CNS	Direct viral infection damages the blood–brain barrier and damages brain tissue, accelerating AD progression.	Neurological complications contributing to AD progression at normal pace.	Kadry et al. (2020)
Neurodegenerative biomarkers	t-tau, p-tau181, GFAP, and NfL significantly increased after COVID-19 infection	Lower when compared to AD patients with COVID-19 (same-age group)	Kadry et al. (2020)

## 6 Conclusion

In conclusion, COVID-19 has generated a worldwide outbreak, resulting in a slew of issues for humans, particularly those suffering from Alzheimer’s disease. Its ability to invade the central nervous system through the hematogenous and neural routes, besides attacking the respiratory system, has the potential to worsen cognitive decline in Alzheimer’s disease patients. Apart from that, the impact of Covid-19 on the brain can be concluded to be like the impact given by Alzheimer’s Disease, as these both cause inflammation. This inflammation, if left untreated, could surely predispose someone who had Covid-19 before to develop Alzheimer’s later, especially if they were seriously infected and experienced long-covid symptoms. Hence, Covid-19 worsens the cognitive decline or impairment in patients with Alzheimer’s and increases the risk of those who had a Covid-19 infection towards developing Alzheimer’s later in life. The severity of this issue must be highlighted.

Moreover, being the most common and prominent type of dementia, Alzheimer’s damage to our cognitive system is beyond our knowledge, and Covid-19 infections further worsen it. Though many research papers are looking at the effects of COVID-19 concerning neurological diseases, such as Alzheimer’s, a definite conclusion has yet to be proven. In this paper, the possible effects of COVID-19 concerning AD, as well as the effects of COVID-19 on AD patients, have been explored. A correlation between the two ailments can be hypothesised. However, still, no finalised answer can be made as to whether one of the aftermath conditions of COVID-19 is a neurological disorder such as Alzheimer’s disease.

## Author contributions

SS: Writing – original draft, Resources. MR: Writing –review & editing, Supervision. SK: Writing – review & editing, Software.
